# Comparison of the impact of two types of removable partial dentures on the periodontal health of the remaining teeth: A prospective clinical study

**DOI:** 10.1002/cre2.738

**Published:** 2023-04-18

**Authors:** Manushaqe S. Bukleta, Mimoza Selmani, Dashnor Bukleta

**Affiliations:** ^1^ College of Medical Science, Faculty of Dentistry “Rezonanca” Pristina Kosovo; ^2^ Dental Clinic Mdent Family Dentistry Pristina Kosovo; ^3^ AAB College ZonaIndustriale Pristina Kosovo

**Keywords:** abutment, oral health, periodontal status, removable partial dentures

## Abstract

**Objective:**

To evaluate and compare the impact of two removable partial dentures (acrylic removable partial denture [ARPD] and metallic removable partial denture [MRPD]) on periodontal tissues of the remaining teeth in the first 12 months of denture use.

**Materials and Methods:**

This prospective clinical study included 40 patients, of which 20 received ARPDs, 20 received MRPDs, nine in the maxilla, and 11 in the mandible each. The patients were 45–65 years old; 24 were females, and 16 were males. Patients’ demographic details, clinical indicators of periodontal complications, and biochemical measurement of Hs‐C‐reactive protein (CRP) and alkaline phosphatase (ALP) were considered. One‐way analysis of covariance and Friedman were used to measure the differences in clinical periodontal parameters between the two types of dentures.

**Results:**

The significant findings were: Plaque index (PLAQ) scores for abutment teeth were higher in MRPD wearers (mean = 12.15) than ARPD wearers (mean = 10.45), whereas ARPD users had significantly higher mean bleeding on probing (BOP) values (mean = 1.5) than MRPD users (mean = 0.00); mobility of abutment teeth showed no significant differences; timeline comparisons showed a significant increase in the percentage of nonabutment teeth mobility in ARPD users (*p* = .028) compared with MRPD users over the same follow‐up period (*p* = .102).

**Conclusions:**

For a short‐term period of 1 year, periodontal and mobility parameters have no significant impact on the abutment and nonabutment teeth of ARPD and MRPD users. Moreover, biochemical markers (CRP and ALP) for periodontal inflammation exhibited no significant difference in both types of dentures.

## 1 INTRODUCTION

Removable partial dentures (RPDs) are the most commonly used removable prosthodontic appliances for partial edentulism. Prosthodontic treatment with RPDs is indicated when fixed prosthodontic treatment is ruled out due to clinical or financial factors. Therefore, it is unsurprising that treatment with RPDs is generally less successful than fixed prosthodontic solutions (Vermeulen et al., 1996). The most common disadvantages of partial dentures are poor oral hygiene, negative impact on the remaining natural teeth, and limited oral comfort (Creugers & de Baat, 2009). However, the proper design of RPD can significantly reduce the incidence of such problems (Fueki et al., 2014). Metal framework dentures have certain advantages over acrylic‐based dentures, for instance, providing a stable denture base and maintenance of oral hygiene (Fueki et al., 2014). Technical and biological complications due to denture wear have been called time‐related issues (Tong et al., 2015). The denture base material may have a critical impact on denture‐supporting tissues. However, acrylic removable partial dentures (ARPDs) may cause problems with the abutment teeth more frequently than Co‐Cr bases, as acrylic is not as rigid as Co‐Cr alloys, making it prone to fracture (Yoshida et al., 2011). One study has shown that ARPDs cause inflammation of gingival tissues more than metallic removable partial dentures (MRPDs) (Bissada et al., 1974). Treatment with ARPDs may result in a rapid loss of the remaining natural teeth and can pose a higher risk for complete edentulism (Allen, 2011).

Biological complications that cause a faster deterioration of the patient's health are more often caused by removable partial dentures than fixed dental dentures (FDPs) (Budtz‐Jørgensen & Isidor, 1990; Thomason et al., 2007). Dentists aim to diagnose periodontal changes that could compromise the long‐term prognosis and therapeutic outcomes (McGivney & Castleberry, 1989). Oral hygiene is even more important for patients with RPDs than their counterparts with fixed appliances (Isidor & Budtz‐Jørgensen, 1990). Since RPDs can cause harmful changes in the quality and quantity of plaque, patients must ensure the highest level of plaque control (Ghamrawy, 1976). One study established that RPDs can increase the risk of dental plaque, gingivitis, and caries but not periodontal disease (Preshaw et al., 2011). Other studies haven't observed any changes in the periodontal parameters in a group of patients with clasp‐retained RPDs, which is interesting since the clasp is generally placed around abutment teeth, resulting in plaque accumulation (Bergman et al., 1995; Ellakwa, 2012).

Periodontitis is characterized by a nonspecific acute‐phase response with acute‐phase reactants like CRP (Chalmers et al., 2008). It was observed that increased C‐reactive protein (CRP) levels persisted among individuals with extensive periodontal diseases (Slade et al., 2000). Also, there is a strong correlation between the intensity of the inflammatory process in periodontal tissues and the level of alkaline phosphatase (ALP) activity (Ainamo et al., 1990).

Periodontal changes in abutment and non‐abutment teeth in both MRPDs and ARPDs have not been assessed. Biochemical markers of periodontal inflammation (CRP and ALP) have never been used to monitor the effects of denture treatment on the human body. Hence, this study aims to evaluate and compare the impact of two removable partial dentures (ARPD and MRPD) on the remaining teeth and periodontal tissues in the first 12 months of denture use.

## 2 MATERIALS AND METHODS

### 2.1 Patient selection and basic data

This prospective study was carried out at the University Dental Clinic of Kosovo, Department for Prosthodontics and Private Dentistry Polyclinic Mdent‐Family Dentistry in Pristina, Kosovo, in cooperation with the Department for Prosthodontics, Faculty of Medicine, University of Ljubljana. The study was conducted between 2016 and 2017 and adhered to the principles outlined in the Declaration of Helsinki (1975). Participation was voluntary, and written consent was obtained. Ethical approval was obtained from the Hospital and University Clinical Service of Kosovo Ethics Committee and the University Clinical Centre of Kosovo (Page No.01 Prot No: 555/18.05.2017). The inclusion criteria were patients aged 45–65, partially edentulous with Kennedy Class I, patients with oral hygiene instructions, and patients needing their first RPD denture. Exclusion criteria were patients with systemic diseases, previously treated with RPD, untreated caries, and disabled persons. Demographic details such as age, gender, level of education, and geographical location were obtained. The condition of the opposite dental arch was noted and classified as (1) complete dental arch with natural teeth with or without fixed prosthodontic treatment; (2) partially edentulous jaw with or without an RPD; and (3) completely edentulous jaw with or without complete dentures.

### 2.2 Clinical and laboratory procedures

Several prosthodontists and dental technicians at the University Dental Clinic of Kosovo, Chair of Prosthodontics and Privat Dentistry Polyclinic Mdent‐Family Dentistry in Pristina, Kosovo, made the RPDs. Twenty‐four participants were females, and 16 were males. Among them, 20 patients received 20 ARPDs, and 20 received 20 MRPDs, 9 maxillary and 11 mandibular each.

**Figure 1:**
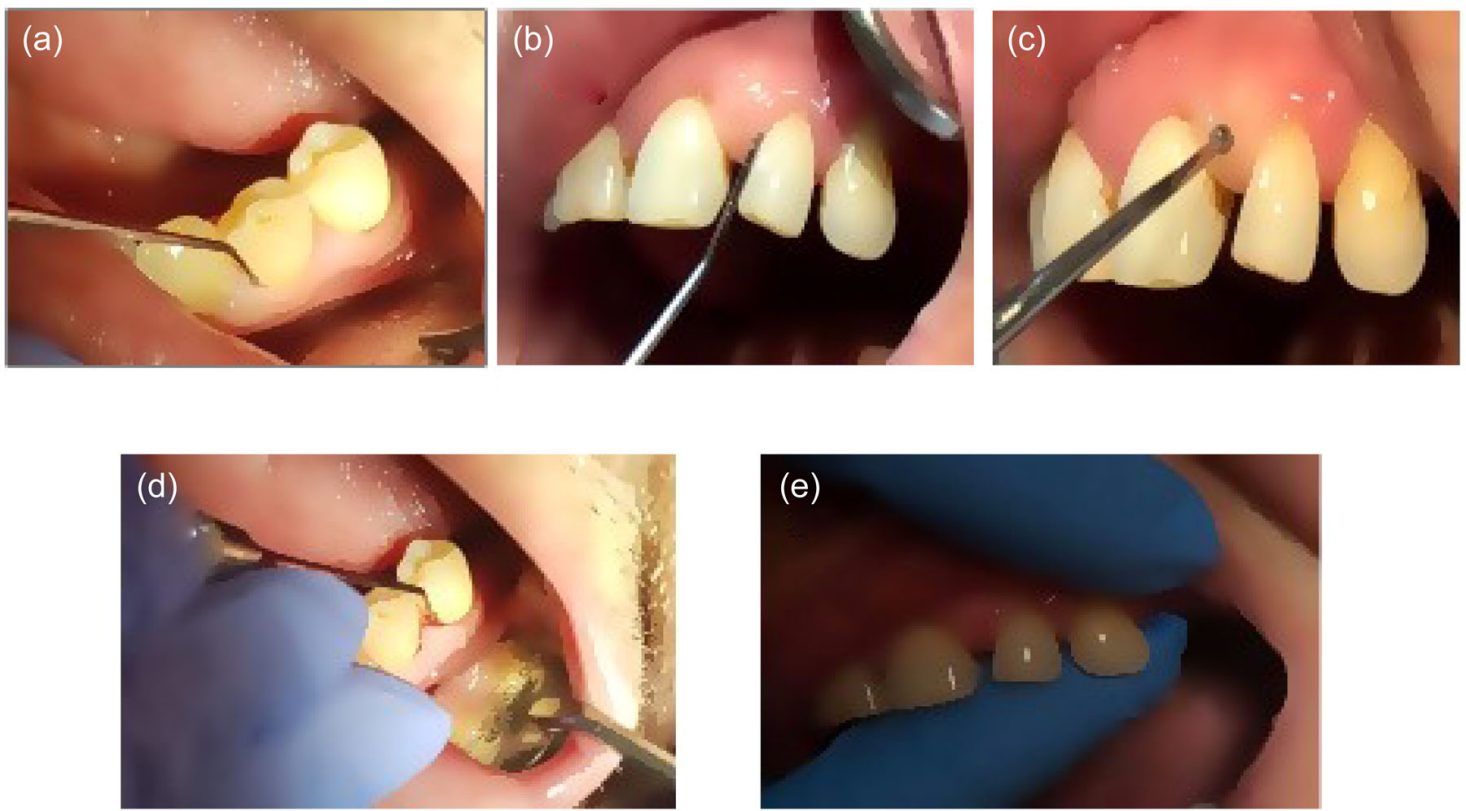
Measurement of periodontal clinical parameters.

The periodontal condition in abutment and non‐abutment teeth of 40 patients was examined through mean probing depth (MPD) and mean attachment level (MAL) expressed in mm, plaque index (PLAQ), and bleeding on probing (BOP) expressed in percentage, and mobility (MOB) at Time 1 (T1), Time 2 (T2), and Time 3 (T3) of wearing RPDs (Figure 1).

– MPD was measured from the gingival margin to the base of the periodontal pocket using a periodontal probe (PCP, UNC‐15, and Hu‐Friedy)
– The clinical attachment level (CAL) was measured from the cementoenamel junction (CEJ) to the base of the pocket.
– The plaque index by Loe and Silness was used (Löe & Silness, 1963).
– Mobility was evaluated using Miller's classification (Miller, 1985).

All periodontal clinical parameters were recorded at six measurement points (mesiobuccal, buccal, distobuccal, mesiolingual, lingual, dand istolingual) in all teeth classified as Kennedy Class I during each of the three visits and were performed and calculated in the online periodontal chart (School of Dental Medicine [ZMK], 2010).

**Figure 2:**
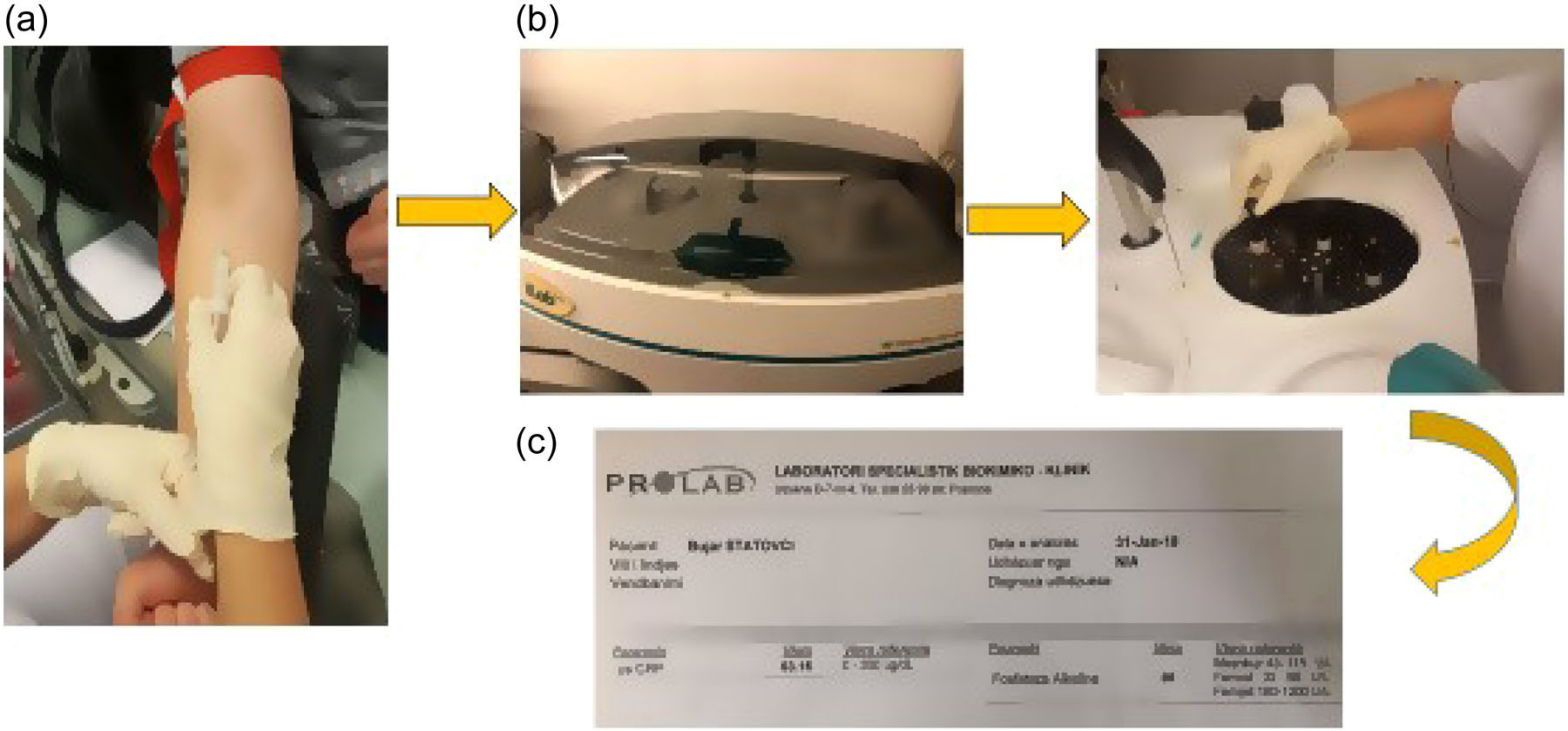
Biochemical analysis: (a) venous blood withdrawal, (b) biochemical analysis, (c) overview of the results.

Periodontal inflammation was evaluated systemically by measuring the content of human serum CRP and ALP at T1, T2, and T3. The instruments used for patient venous blood collection were: medical syringes (Sarsedt Monovette Clot Activator 7.5 mL), latex gloves, and an Esmarch bandage (100–150 cm). Hs‐CRP was measured with a latex immunoturbidimetric assay (Quantex CRP Ultrasensitive reagent, BIOKIT), while ALP was measured with the enzymatic method from International Federation of Clinical Chemistry (IFCC) with p‐nitrophenylphosphate at 37 C. Both hs‐CRP and ALP were measured by Instrumentation Laboratory Company ILAB ARIES 2014, Milano, Italy, in the biochemical laboratory PROLAB in Pristina, Kosovo. The procedure of the biochemical analysis is shown in Figure 2.

### 2.3 Statistical analysis

The statistical analysis was performed using SPSS software version 22.00. Statistical significance was established at *α* = .05. Descriptive statistics were used for patient data and the prosthodontic treatment status of the dental arch opposite the treated jaw. Friedman test was employed for biochemical parameters (CRP and ALP) along with paired *t*‐test. Periodontal parameters employed two approaches: The first was to analyse the time changes of clinical periodontal measurements for two groups of patients: ARPDs patients and MRPDs patients. The second approach compared MRPDs and ARPDs to periodontal measurements through one‐way analysis of covariance to determine significant differences in each. The comparison of clinical periodontal and biochemical paramteres (CRP and ALP) for the abutment and non‐abutment teeth was carried out with the One‐way analysis of covariance test for the two types of dentures at three different time points (T1, T2, and T3).

## 3 RESULTS

The study was carried out on a sample of 40 patients—24 females (60%) and 16 males (40%)—with a mean age of 53.6 years (±8.60), of which 32.5% were living in rural areas and 67.5% in urban areas. The frequency distribution of the patients’ education, type of denture, and antagonist jaw are presented in Table 1.

**Table 1:** Descriptive statistics and frequency distribution of various variables.

Variables (*N* = 40)	Mean (Std. dev)	Frequency	Percentage (%)
*Age*	53.63 (±8.60)		
Gender			
Female		24	60.0
Male		16	40.0
Region			
Rural		13	32.5
Urban		27	67.5
Education			
<4 years		1	2.5
5–8 years		18	45.0
9–12 years		20	50.0
>12 years		1	2.5
Type of denture			
MRPD		20	50
ARPD		20	50
Jaw and type of denture			
MRPD upper		9	22.5
ARPD upper		9	22.5
MRPD lower		11	27.5
ARPD lower		11	27.5
Antagonist Jaw			
Complete denture		3	7.5
Partial denture		15	37.5
Subtotal denture		5	12.5
Natural teeth with fixed restoration		8	20.0
Natural teeth		9	22.5

Cross‐tabulation of the type of denture and antagonist jaw shows the percentage of patients with MRPDs and ARPDs that received prosthodontics treatment or had natural teeth in their antagonist jaw (Table 2).

**Table 2:** Descriptive statistics showing cross‐tabulation of the type of denture and antagonist jaw.

			Antagonist jaw					Total
			Complete denture	Partial denture	Subtotal denture	Fixed restorations	Natural teeth	
Type of denture	MRPD	*f*	0	8	0	6	6	20
		*f*%	0.0%	40.0%	0.0%	30.0%	30.0%	100.0%
	ARPD	*f*	3	7	5	2	3	20
		*f*%	15.0%	35.0%	25.0%	10.0%	15.0%	100.0%
Total		*f*	3	15	5	8	9	40
		*f*%	7.5%	37.5%	12.5%	20.0%	22.5%	100.0%

Abbreviations: ARPDs, acrylic removable partial dentures; MRPDs, metallic removable partial dentures.

**Figure 3:**
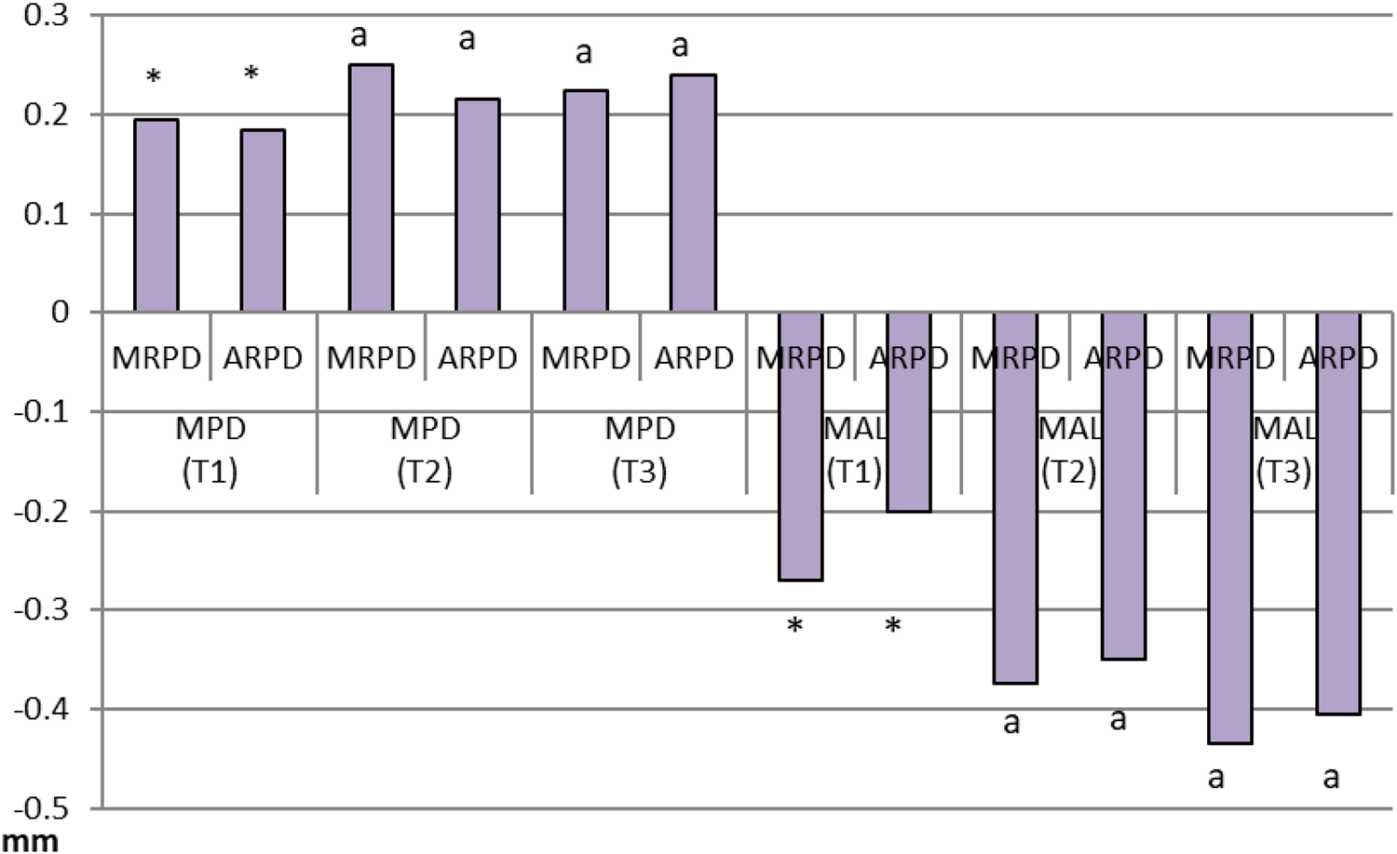
Mean MPD and MAL scores for patients with MRPDs and ARPDs at three‐time intervals (abutment teeth). ARPDs, acrylic removable partial dentures; MAL, mean attachment level; MPD, mean probing depth; MRPDs, metallic removable partial dentures.

**Figure 4:**
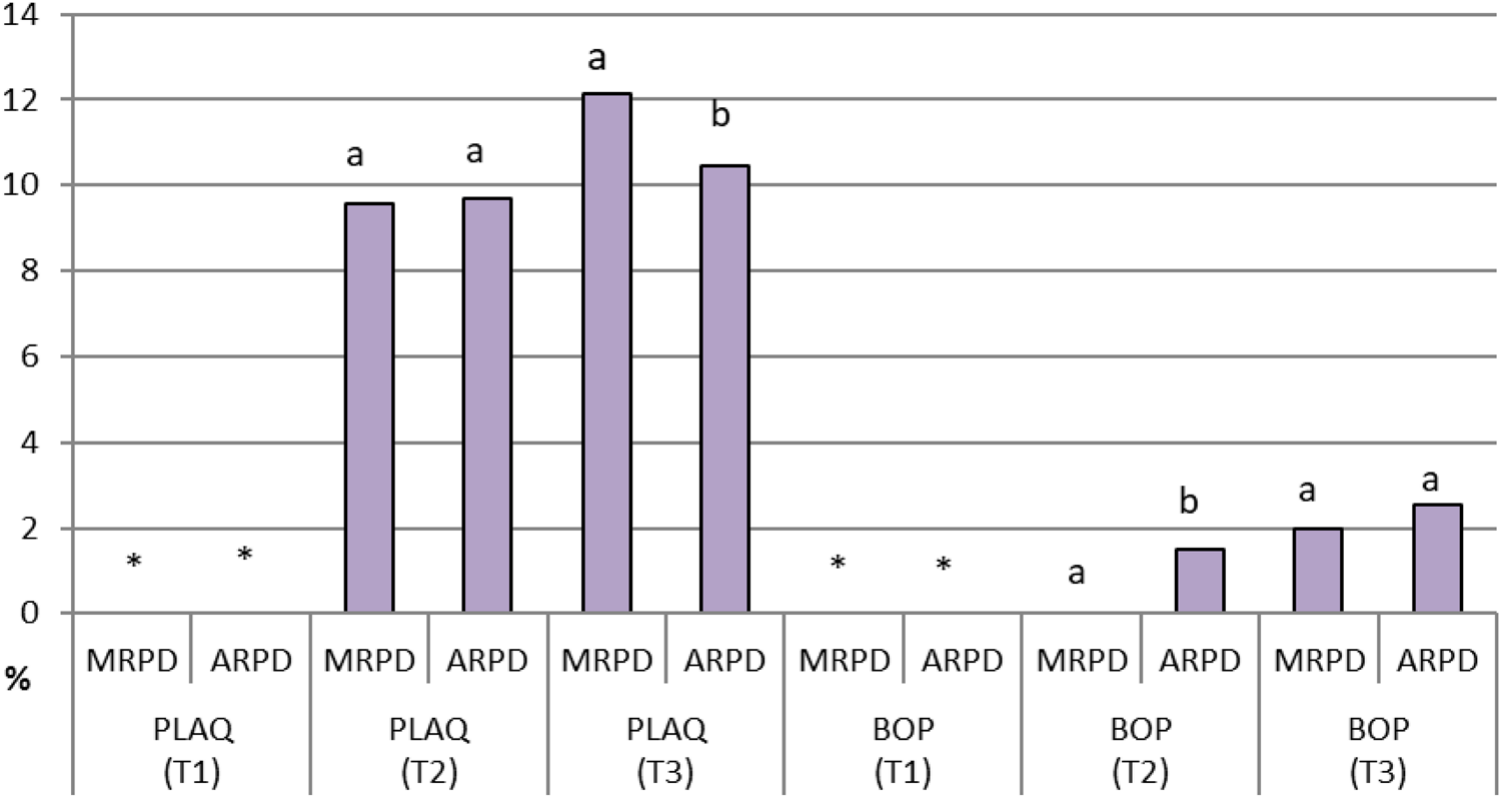
Mean PLAQ and BOP scores for patients with MRPDs and ARPDs presented at three‐time intervals (abutment teeth). ARPDs, acrylic removable partial dentures; BOP, bleeding on probing; MRPDs, metallic removable partial dentures; PLAQ, plaque index.

Regarding the periodontal parameters for abutment teeth, patients with MRPDs had significantly higher PLAQ values (mean = 12.15) than their ARPD counterparts (mean = 10.45). ARPD users had significantly higher mean BOP values (adj. mean = 1.5) than MRPD users (adj. mean = 0.00) (Table 3, Figures 3 and 4).

**Table 3:** Comparison of periodontal parameters of abutment teeth at T2 and T3 after T1.

Periodontal parameters at each time point	Type of denture	*N*	Mean	Mean (including covariances)	Std. deviation	*F*	*p*
MPD (T1)	MRPD	20	0.195	/	0.076	/	/
	ARPD	20	0.185	/	0.049		
MPD (T2)	MRPD	20	0.250	0.248	0.061	3.281	.078
	ARPD	20	0.215	0.217	0.059		
MPD (T3)	MRPD	20	0.225	0.223	0.064	1.112	.298
	ARPD	20	0.240	0.242	0.068		
MAL (T1)	MRPD	20	−0.270	/	0.098	/	/
	ARPD	20	−0.200	/	0.138		
MAL (T2)	MRPD	20	−0.375	−0.367	0.097	0.089	.767
	ARPD	20	−0.350	−0.358	0.089		
MAL (T3)	MRPD	20	−0.435	−0.427	0.109	0.144	.707
	ARPD	20	−0.405	−0.413	0.105		
PLAQ (T1)	MRPD	20	0.000	/		/	/
	ARPD	20	0.000	/			
PLAQ (T2)	MRPD	20	9.550	9.55	5.216	0.012	.914
	ARPD	20	9.700	9.70	3.246		
**PLAQ** (T3)	MRPD	20	12.150	12.15	2.834	4.136	**.049**
	ARPD	20	10.450	10.45	2.439		
BOP (T1)	MRPD	20	0.000	/		/	/
	ARPD	20	0.000	/			
**BOP** (T2)	MRPD	20	0.000	0.000	0.000	4.622	**.038**
	ARPD	20	1.500	1.500	3.120		
BOP (T3)	MRPD	20	2.000	2.000	2.534	.281	.599
	ARPD	20	2.550	2.550	3.887		

*Note*: One‐way analysis of covariance test.

Abbreviations: ARPDs, acrylic removable partial dentures; BOP, bleeding on probing; MAL, mean attachment level; MPD, mean probing depth; MRPDs, metallic removable partial dentures; PLAQ, plaque index.

**Figure 5:**
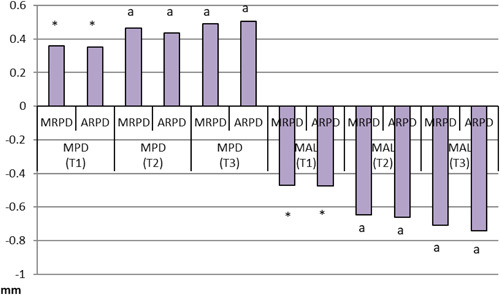
Mean MPD and MAL scores for patients with MRPDs and ARPDs presented at three‐time intervals (non‐abutment teeth). ARPDs, acrylic removable partial dentures; MAL, mean attachment level; MPD, mean probing depth; MRPDs, metallic removable partial dentures.

**Figure 6:**
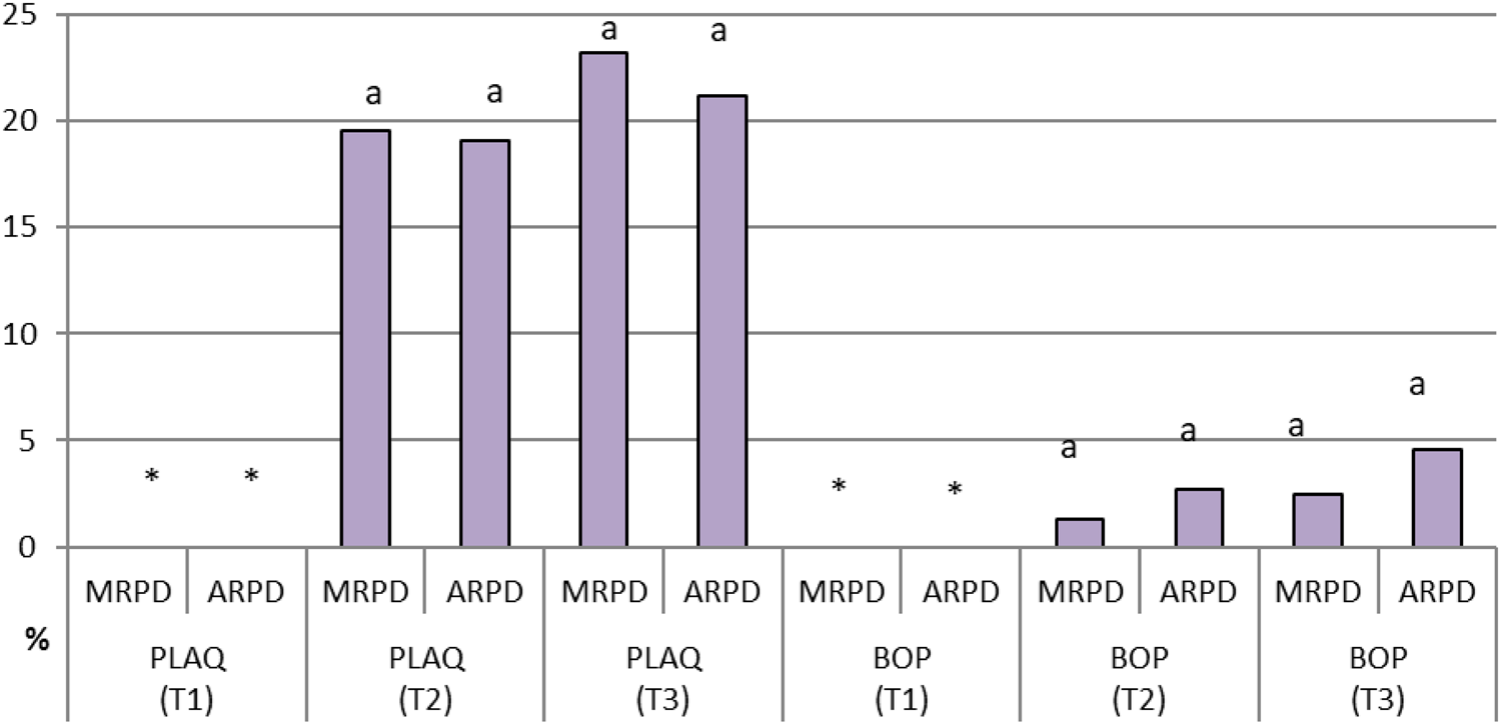
Mean PLAQ and BOP scores for patients with MRPDs and ARPDs at three‐time intervals (non‐abutment teeth). ARPDs, acrylic removable partial dentures; BOP, bleeding on probing; MRPDs, metallic removable partial dentures; PLAQ, plaque index.

Periodontal parameters for non‐abutment teeth did not vary significantly between the two types of dentures at three different intervals. The recorded values for various periodontal parameters are in Table 4, Figures 5 and 6.

**Table 4:** Comparison of periodontal parameters of nonabutment teeth in patients with MRPDs and ARPDs between T1 and T2 or T3.

Periodontal parameters at each time point	Type of denture	*N*	Mean	Mean (including covariances)	Std. Deviation	*F*	*p*
MPD (T1)	MRPD	20	0.360	/	0.105		
	ARPD	20	0.350	/	0.115		
MPD (T2)	MRPD	20	0.465	0.461	0.150	0.376	.544
	ARPD	20	0.435	0.439	0.139		
MPD (T3)	MRPD	20	0.490	0.487	0.125	0.262	.612
	ARPD	20	0.505	0.508	0.157		
MAL (T1)	MRPD	20	−0.470	/	0.223		
	ARPD	20	−0.475	/	0.205		
MAL (T2)	MRPD	20	−0.645	−0.647	0.254	0.033	.857
	ARPD	20	−0.660	−0.658	0.282		
MAL (T3)	MRPD	20	−0.710	−0.712	0.234	0.146	.705
	ARPD	20	−0.740	−0.738	0.298		
PLAQ (T1)	MRPD	20	0.000	/	0.000		
	ARPD	20	0.000	/	0.000		
PLAQ (T2)	MRPD	20	19.55	19.55	10.200	.026	.874
	ARPD	20	19.10	19.10	7.391		
PLAQ (T3)	MRPD	20	23.20	23.20	5.146	1.286	.264
	ARPD	20	21.15	21.15	6.235		
BOP (T1)	MRPD	20	0.000	/	0.000		
	ARPD	20	0.000	/	0.000		
BOP (T2)	MRPD	20	1.30	1.30	3.373	.678	.415
	ARPD	20	2.70	2.70	6.814		
BOP (T3)	MRPD	20	2.45	2.45	4.161	1.981	.167
	ARPD	20	4.55	4.55	5.216		

*Note*: One‐way analysis of covariance test.

Abbreviations: ARPDs, acrylic removable partial dentures; BOP, bleeding on probing; MAL, mean attachment level; MPD, mean probing depth; MRPDs, metallic removable partial dentures; PLAQ, plaque index.

In (Table 5) the percentage of mobile teeth and the percentages of time‐point mobility for each type of denture (MRPDs and ARPDs) have been noted, and in (Table 6) obtained results for two kinds of dentures were compared using one‐way analysis of covariance.

**Table 5:** Time comparison of abutment teeth mobility in patients with MRPDs and ARPDs.

Teeth mobility per type of denture (%)	Time point	*N*	Mean	Median	Standard deviation	U	*p*
MOB (MRPD)	T1	20	0.00	0.00	0.00	4.667	.097
	T2	20	5.00	0.00	22.36		
	T3	20	9.65	0.00	25.65		
MOB (ARPD)	T1	20	0.00	0.00	0.00	2.000	.368
	T2	20	2.50	0.00	11.18		
	T3	20	2.50	0.00	11.18		

Abbreviations: ARPDs, acrylic removable partial dentures; MOB, mobility; MRPDs, metallic removable partial dentures.

**Table 6:** Comparison of abutment tooth mobility in patients with MRPDs and ARPDs at T1, T2, and T3.

Teeth mobility at each time point (%)	Type of denture	*N*	Mean	Mean (Adjusted)	Standard deviation	F	*p*
MOB (T1)	MRPD	20	0.00	/	0.00	/	/
	ARPD	20	0.00	/	0.00		
MOB (T2)	MRPD	20	5.00	5.00	22.36	0.200	.657
	ARPD	20	2.50	2.50	11.18		
MOB (T3)	MRPD	20	9.65	9.65	25.65	1.296	.262
	ARPD	20	2.50	2.50	11.18		

*Note*: One‐way analysis of covariance test.

Abbreviations: ARPDs, acrylic removable partial dentures; MOB, mobility; MRPDs, metallic removable partial dentures.

The percentage of mobile nonabutment teeth was calculated, and percentages of time‐based mobility for each type of denture were presented (Table 7).

**Table 7:** Time comparison of the mobility of the non‐abutment teeth for patients wearing MRPDs and ARPDs.

Teeth mobility per type of denture %	Time point	*N*	Mean	Median	Standard deviation	*χ* ^2^	*p*
MOB (MRPD)	T1	20	0.0	0.0	0.0	4.667	.097
	T2	20	2.5	0.0	11.18		
	T3	20	7.14	0.0	17.50		
MOB (ARPD)	T1	20	0.0	0.0	0.00	9.579	**.008**
	T2	20	8.96	0.0	20.93		
	T3	20	16.11	0.0	26.51		

Abbreviations: ARPDs, acrylic removable partial dentures; MOB, mobility; MRPDs, metallic removable partial dentures.

Hs‐CRP and ALP levels were measured for both types of dentures at three different time intervals. The results for patients with MRPDs did not show any statistically significant differences in ALP values at the three‐time intervals (*p*‐value = .116) (Table 8), and similar results were obtained for CRP values (*p*‐value = .387). However, a post hoc analysis revealed a slightly significant increase in CRP values from 178.29 to 290.93 (*p*‐value = .048) at T2 (Table 9).

**Table 8:** Time comparison of biochemical parameters in patients with MRPDs and ARPDs.

Biochemical parameters per denture	Time point	*N*	Mean	Median	Std. Deviation	*F*/*χ* ^2^	*p*
ALP (MRPD)	T1	20	92.64	94.00	22.10	4.300	.116
	T2	20	90.11	88.50	24.69		
	T3	20	98.71	76.00	53.93		
ALP (ARPD)	T1	20	102.66	98.00	32.81	4.582	.101
	T2	20	100.66	81.50	57.94		
	T3	20	88.22	74.50	34.75		
CRP (MRPD)	T1	20	178.29	175.15	127.08	1.900	.387
	T2	20	290.93	234.70	237.28		
	T3	20	284.80	236.50	231.17		
CRP (ARPD)	T1	20	204.15	154.00	143.10	0.684	.711
	T2	20	313.72	221.95	270.88		
	T3	20	258.25	149.50	326.77		

*Note*: Friedman test.

Abbreviations: ALP, alkaline phosphatase; ARPDs, acrylic removable partial dentures; CRP, C‐reactive protein; MRPDs, metallic removable partial dentures.

**Table 9:** Pairwise time comparison with a post hoc analysis of biochemical parameters in patients with MRPDs and ARPDs.

Biochemical parameters per type of denture	Pairwise time comparison	*N*	*t*/*Z*	*p*
ALP (MRPD)	T1–T2	20	−0.485^b^	.627
	T1–T3	20	−0.523^b^	.601
	T2–T3	20	−0.112^b^	.911
ALP (ARPD)	T1–T2	20	−0.710^b^	.478
	T1–T3	20	1.840^a^	.081
	T2–T3	20	−0.803^b^	.422
CRP (MRPD)	T1–T2	20	−1.979^b^	**.048**
	T1–T3	20	−1.718^b^	.086
	T2–T3	20	−0.859^b^	.390
CRP (ARPD)	T1–T2	20	−1.530^b^	.126
	T1–T3	20	−0.075^b^	.940
	T2–T3	20	−1.382^b^	.167

Abbreviations: ALP, alkaline phosphatase; ARPDs, acrylic removable partial dentures; CRP, C‐reactive protein; MRPDs, metallic removable partial dentures.

^a^ Paired sample *t*‐test,

^b^ Wilcoxon signed‐rank test.

The One‐way analysis of covariance was performed to assess the differences in ALP and CRP levels between both types of dentures after a particular time. Covariates were incorporated, and the adjusted means were compared. The mean values were adjusted according to the initial values, and the outliers were deleted from the data set. The assumption for homogeneity was checked with Levene's test for homogeneity of variance, and the interaction between the covariate and the independent variable, if any, was assessed. In all the cases, the assumption of homogeneity of regression slopes was met (Table 10).

**Table 10:** Comparison of biochemical parameters in patients with ARPDs and MRPDs at T1, T2, and T3.

Biochemical parameters	Type of denture	*N*	Mean	Mean (adjusted)	Std. Deviation	Levene's test	Homogeneity	*F*	*p*
ALP(T1)	MRPD	20	92.64	/	22.10			/	/
	ARPD	20	102.6	/	32.81				
ALP(T2)	MRPD	20	90.11	90.615	24.69	0.497	0.280	1.257	.270
	ARPD	18	82.96	82.394	20.52				
ALP(T3)	MRPD	19	88.74	91.672	31.22	0.600	0.512	0.426	.518
	ARPD	20	88.22	85.432	34.75				
CRP(T1)	MRPD	20	178.29	/	127.08			/	/
	ARPD	20	204.15	/	143.10				
CRP(T2)	MRPD	20	290.93	307.254	237.28	0.755	0.197	0.026	.872
	ARPD	20	313.72	297.386	270.88				
CRP(T3)	MRPD	20	284.80	291.345	231.17	0.017	0.840	0.462	.083
	ARPD	19	195.82	188.932	174.42				

*Note*: One‐way analysis of covariance.

Abbreviations: ALP, alkaline phosphatase; ARPDs, acrylic removable partial dentures; CRP, C‐reactive protein; MRPDs, metallic removable partial dentures.

## 4 DISCUSSION

A removable partial denture remains the most common treatment for partially edentulous jaws or severely compromised dentition. The main goal of a prosthodontic appliance is to restore the esthetics and function of the oral cavity without further damage to the oral tissues. In our study, analysis of periodontal parameters performed between the abutment teeth of MRPD and ARPD wearers revealed no significant difference between MPD and MAL measurements at various time intervals. A significant difference was observed between the PLAQ scores, where in MRPD patients, mean scores (mean = 12.15) were higher compared with the patients wearing an ARPD (mean = 10.45). Furthermore, a difference was observed in BOP scores where ARPD users had significantly higher values (2.55) when compared to their MRPD counterparts (2.00). These results match the study conducted in Kosovo, where there was an increase in the mean scores of BOP, PD, and plaque index on the abutment teeth three months following RPD insertion (Dula et al., 2015). Another study observed an increased prevalence of plaque, gingival recession, and gingivitis, especially on dentogingival surfaces within 3 mm proximity to the dentures (Yeung et al., 2000). Furthermore, a study by Amaral et al. (2010) concluded that plaque scores, mean probing depth, and gingival index score had increased significantly in abutment teeth compared to non‐abutment teeth at a follow‐up period of 3 months.

Periodontitis is characterized by a pathological deepening of the gingival sulcus. Our study shows a slight increase in clinical periodontal measurements in both abutment and nonabutment teeth observed across both types of RPDs. A study showed that high plaque scores and a maintenance interval longer than 3 months were significant predictors for changes observed in the periodontium (do Amaral et al., 2010). Another concluded that in patients with removable partial dentures, the direct abutment teeth were periodontally affected compared to non‐abutment teeth among regular and irregular attendants over 3 years (Qudah & Nassrawin, 2004).

It was found that patients wearing an ARPD or MRPD did not have any significant increase in mobility on their abutment teeth over 12 months. These results are concomitant with the study of Ammara et al. (2010). The results for ARPD wearers indicate a significant increase in patients’ tooth mobility between T1 and T3, while MRPD wearers showed no statistical increase in tooth mobility. Similar results were seen in a study where tooth mobility was relatively unchanged in abutment teeth with a follow‐up of 5 years. It was observed that non‐abutment teeth showed an initial decline and then significantly improved throughout the follow‐up (Qudah & Nassrawin, 2004).

Biochemical markers like CRP and ALP have been used as valuable adjuncts for diagnosing periodontitis (Chiba et al., 2005; Izuora et al., 2016). The timeline comparisons of ALP and CRP levels for both types of dentures did not reveal any statistical significance, as there was no definitive trend to their levels throughout the follow‐up. The pairwise comparison of the periods showed that CRP levels from T1 to T2 in MRPDs had statistical significance, and the individual comparisons of patients with ARPDs or MRPDs did not show any statistical importance. This may be attributed to the fact that a surface layer of protein typically develops on metallic dentures and ensures biocompatibility in titanium dentures. However, this acts as a double‐edged sword, as it may aid in the colonization of micro‐organisms, thereby allowing biofilm formation.

The major limitation of this study is the short follow‐up period. However, this study can pave the way for future research on the complications between different dentures. It also provides tools to help monitor and analyse oral health indicators and the short‐term effects of denture wear.

## 5 CONCLUSION

During the 12‐month investigation, we concluded that ARPDs and MRPDs carry almost the same risk. Therefore, the choice to use ARPDs as a temporary removable prosthodontic treatment for up to 1 year is fully justified.

## AUTHOR CONTRIBUTIONS

Manushaqe Selmani Bukleta wrote the main manuscript text. Mimoza Selmani helped in patients selection and examination. Dashnor Bukleta worked on the statistic analysis of the research. All authors reviewed the manuscript.

## CONFLICT OF INTEREST STATEMENT

All authors declare no competing interests.

## ETHICS STATEMENT

Ethical approval was obtained from the Hospital and University Clinical Service of Kosovo Ethics Committee and the University Clinical Centre of Kosovo (Page No.01 Prot No: 555/18.05.2017).

## PATIENT CONSENT

Patient's written consent was taken before administering the questionnaire and clinical examination.

## Data Availability

Additional data supporting these findings are provided in this article's supplementary material.
